# Human marrow stromal cells reduce microglial activation to protect motor neurons in a transgenic mouse model of amyotrophic lateral sclerosis

**DOI:** 10.1186/1742-2094-10-52

**Published:** 2013-04-30

**Authors:** Chang Zhou, Chen Zhang, Renliang Zhao, Song Chi, Ping Ge, Cheng Zhang

**Affiliations:** 1 Department of Neurology, The Affiliated Hospital of Medical College, Qingdao University, 16 Jiangsu Road, Qingdao, Shandong 266003, PR China; 2 Department of Neurology, The First Affiliated Hospital of Sun Yat-Sen University, 58 Zhongshan Second Road, Guangzhou, Guangdong 510080, PR China

**Keywords:** Amyotrophic lateral sclerosis, Bone marrow stromal cells, Cu/Zn superoxide dismutase 1, Intrathecal transplantation, Microglia

## Abstract

**Background:**

Human marrow stromal cells (hMSCs) injected intrathecally can effectively increase the lifespan and protect motor neurons in a transgenic mouse model of amyotrophic lateral sclerosis. However, how the transplanted cells exert a neuroprotective effect is still unclear. More recently, the anti-inflammation effect of marrow stromal cells has generated a great deal of interest. In the present study, we sought to investigate whether intrathecally injected hMSCs protect motor neurons through attenuating microglial activation and the secretion of inflammatory factors in Cu/Zn superoxide dismutase 1 (SOD1) transgenic mice. In addition, we also focused on the mode of hMSCs inhibiting microglial activation.

**Methods:**

We transplanted hMSCs into the cisterna magna of SOD1 mice at the age of 8, 10 and 12 weeks. At sacrifice, tissues were harvested for analysis of neuron counts, microglial activation, TNFα secretion and inducible nitric oxide synthase (iNOS) protein expression. *In vitro*, microglial cells were treated with hMSC co-culture, hMSC transwell culture or hMSC conditioned medium to investigate the mode of hMSCs exerting an anti-inflammation effect.

**Results:**

Intrathecally transplanted hMSCs inhibited inflammatory response in SOD1 transgenic mice, which was evidenced by the decreases in microglial activation, TNFα secretion and iNOS protein expression. In addition, the inhibitory effect on microglial activation of hMSCs was through secretion of diffusible molecules adjusted to environmental cues.

**Conclusion:**

Intrathecally injected hMSCs can attenuate microglial activation through secretion of diffusible molecules to exert a therapeutic effect in SOD1 transgenic mice. Further studies are needed to explore the exact mechanisms by which hMSCs inhibit inflammation for facilitating the therapeutic effect.

## Background

Amyotrophic lateral sclerosis (ALS) is a fatal neurodegenerative disorder characterized by progressive loss of spinal cord and cortical motoneurons. A transgenic mouse model expressing the human G93A mutant variant of Cu/Zn superoxide dismutase 1 (SOD1) is a well-accepted model of the disease [1]. Stem cell transplantation is a promising therapeutic strategy for ALS. The functional replacement of lost motoneurons is proved to be very difficult, and more likely transplanted cells exert their neuroprotective effect.

Human marrow stromal cells (hMSCs) are isolated from adult bone marrow, and easy access suggests their feasibility in clinical therapies. Marrow stromal cells (MSCs) have the potential to differentiate to lineages of mesenchymal tissues such as bone, cartilage, fat, tendon and muscle [2]. A rare cell type within MSCs called multipotent adult progenitor cells, similar to embryonic stem cells, have been identified [3]. MSCs express a variety of cytokines and trophic factors [4]. The role of MSCs as nurse cells supporting the function of other cell types in bone marrow suggests their potential use as neuroprotective cells. Moreover, autologous transplantation of these cells would circumvent potential ethical and immunological concerns. The advantages suggest that hMSCs may be suitable candidates for human therapies. Our previous studies showed that multiple intrathecal administration of hMSCs effectively increased the lifespan and protected motorneurons in SOD1 mice, although very few injected hMSCs were detected in the parenchyma of spinal cord [5]. However, it is still unclear how the cells injected intrathecally exert a neuroprotective effect.

Activated microglia and proinflammatory cytokines including TNFα secreted by these cells appeared to play an important role in disease progression of SOD1 transgenic mice [6]. Moreover, encouraging preliminary results have been obtained in SOD1 transgenic mice with the cyclooxygenase-2 inhibitor, with which a 20% prolongation of survival was reported [7]. Targeting therapy to microglia may therefore be an effective strategy for ALS. More recently, the anti-inflammation effect of MSCs as a protective mechanism has been demonstrated *in vitro*[8-10] and in animal models of Parkinson disease [11], cerebral ischemia [12,13], Krabbe’s disease [14] and ALS [15], which has generated a great deal of interest. Locally injected hMSCs in the lumbar spinal of SOD1 mice reduced microglial activation and reactive astrocytosis [15], but whether the intrathecally injected hMSCs inhibit microglial activation and attendant secretion of inflammatory factors remains unclear.

In the present study, we sought to investigate whether intrathecally injected hMSCs protect motor neurons by attenuating microglial activation and inflammatory factor secretion in SOD1 transgenic mice. In addition, we also focused on the mode of hMSCs inhibiting microglial activation.

## Methods

### Animals

Transgenic mice of the strain B6SJL-TgN (SOD1-G93A) 1Gur, which harbor human SOD1 with the G93A mutation in high copy number, were obtained from Jackson Laboratory (Bar Harbor, Maine, USA). We maintained the transgenic G93A hemizygotes by mating transgenic males with B6SJLF1/J hybrid females. Transgenic mice were identified by PCR using primers and a protocol suggested by the vendor. All mice were housed in the specific pathogen-free animal facility of Sun Yat-Sen University, Guangdong, PR China. From the time when transgenic mice showed motor deficits, nutritional gel was routinely placed in the cages of all transgenic animals for easy access to food and hydration. Transgenic mice were randomly divided into two groups: Group 1, hMSC-treated group – mice (*n* = 27) were intrathecally injected with hMSCs at the age of 8, 10 and 12 weeks; Group 2, vehicle-treated group – mice (*n* = 29) were administered PBS at the age of 8, 10 and 12 weeks. Age-matched wild-type littermate mice (*n* = 25) were used as the normal control group. Experiments were conducted in accordance with protocols approved by the Animal Care and Use Committee of Sun Yat-Sen University. Ethical Approval was given by Sun Yat-Sen University Research Ethics Committee (reference number: ZS09072).

### Preparation of human marrow stromal cells

After obtained informed consent, 2 ml bone marrow was aspirated from the iliac crest of healthy normal volunteers. This protocol was approved by the Institutional Review Board of Sun Yat-Sen University. Mononuclear cells were isolated by Ficoll density gradient centrifugation (1.077 g/ml; Sigma, Munich, Germany) at 400× *g* for 35 minutes. The mononuclear cells were resuspended in the culture medium composed of DMEM (GIBCO, Rockville, MD, USA) with low glucose and 10% fetal bovine serum (FBS; Hyclone, Logan, UT, USA). The mononuclear cells were plated at 1×10^6^ cells/25 cm^2^ in culture flasks and the cultures were incubated at 37°C in 5% CO_2_ in air and 95% humidity. The medium was exchanged after 48 hours and every 3 to 4 days thereafter. When the cultures reached approximately 90% of confluence, hMSCs were passaged with 0.25% trypsin (GIBCO) and replated into passage culture at a density of 5,000 to 10,000 cells/cm^2^. Cells at passages 3 and 4 were used for transplantation. Upon harvest, cells were isolated by treatment with 0.25% trypsin. The cells were then washed four times and resuspended in PBS at a cellular density of 100,000 cells/μl. Viability of cells was assessed using a 0.4% Trypan Blue dye (Sigma) exclusion method prior to and following transplantation. The cells were analyzed for their immunophenotype by flow cytometry (Beckman Coulter, Fullerton, CA, USA). Fluorescein isothiocyanate (FITC)-conjugated or phycoerythrin-conjugated antibodies specific for human CD14, CD19, CD45, CD34, CD73, CD90, CD105 and HLA-DR tested for flow cytometry application. The percentage of positive cells was determined based on the fluorescent emission of the nonspecific FITC/phycoerythrin isotypic antibody controls.

### Transplantation

Transgenic mice were anesthetized with pentobarbital (60 mg/kg, intraperitoneally), and were placed in a prone position with the head flexed below horizontal by approximately 30°. The posterior scalp was incised in the midline and the muscles were separated by blunt dissection with forceps. At the junction of the occipital bone and atlas, the dura mater was exposed through which a blood vessel can be seen (arteria dorsalis spinals). The atlanto-occipital membrane was blotted dry and pierced with a Hamilton syringe and 30-gauge needle 45° caudally from the middle toward the left side. Cerebrospinal fluid was aspirated to make sure that the tip of the needle was in the cisterna magna. Then 5 μl cell suspension (approximately 5×10^5^ hMSCs) or 5 μl vehicle (PBS) was injected into the cisterna magna slowly over 5 minutes. All animals were immunosuppressed with cyclosporine (10 mg/kg, intraperitoneally, per day) 3 days before surgery and thereafter.

### Assessment of disease progression

Beginning at 8 weeks of age, the mice were monitored for disease onset and progression weekly by a well-trained experienced observer blinded to the genotype. We used testing methods including simple clinical observation and the hanging wire test. Onset was defined retrospectively as the earliest time when the mice showed symptoms (tremulousness and/or gait abnormalities) for ≥2 consecutive weeks. Because of ethical considerations, transgenic animals were euthanized when they were unable to right themselves within 30 seconds, and this time point was recorded as the time of death.

The hanging wire test evaluates paw grip endurance. The mouse was placed on a wire cage lid and the lid was gently waved in the air so that the mouse griped the wire. The lid was then turned upside down approximately 50 cm above the surface of soft bedding material. The latency until a mouse let loose with both hind limbs was recorded, with a cutoff time of 90 seconds. Each mouse was given three consecutive trials, and the longest latency was recorded.

### Spinal cord history and neuron counts

The mice (*n* = 4 each) at the ages of 11 weeks (a time point of presymptom) and 15 weeks (a time point of full symptoms) were killed under deep chloral hydrate (10%) anesthesia. Mice were intracardially perfused with 0.1 M PBS (pH 7.4) for 1 minute, followed by cold 4% paraformaldehyde in PBS (pH 7.4) for 10 minutes. The spinal cords were dissected carefully, and the lumbar segment was identified using the ribs and the vertebrate as a guide. Tissues were postfixed in 4% paraformaldehyde for 6 hours. Blocks were cryoprotected in 30% sucrose for 24 hours, and then were embedded transversely in OCT compound (Tissue Tek®, Elkhart, IN, USA). They were then serially sectioned for 2 mm of the lumbar enlargement at 10 μm on a cryostat. Sections were mounted on gelatinized glass slides and stored at −20°C. A set of sections included 15 sections in total (every fifth section to avoid false double-counting) from each animal. Both right and left hind limbs were studied and the results were averaged.

For neuron counts, a section was stained with 1% cresyl violet. Cell counts were made within an area demarcated by a horizontal line drawn through the central canal and encompassing the ventral horn of grey matter to include layers 7 to 9. Each section was visualized with an light microscope (Olympus B×51, Tokyo, Japan) at 200× magnifications, captured onto a computer using Image-pro-plus 5.1(Media Cybernetics Inc, Silver Spring, MD, USA) and counted manually by tracing the perimeter of each motor neuron. We identified motoneurons and analyzed them for cell area using the following criteria [7]: the presence of a large single nucleolus located within the nucleus, surrounded by light blue staining cytoplasm; and a cell somal area over 100 μm^2^. Gamma motor neurons range from 100 to 250 μm^2^, whereas the larger α motor neurons range from 250 to 1,100 μm^2^.

For immunohistochemistry, sections were permeabilized in 0.3% Triton X-100, blocked with normal serum, and then incubated with primary antibody at 4°C overnight. Primary antibodies included: rat anti-mouse CD11b (1:50 dilution; Chemicon, Temecula, CA, USA). Sections were washed and incubated at room temperature for 1 hour with goat anti-rat FITC-conjugated IgG (1:50 dilution; Serotec, Raleigh, NC, USA). All staining involved the use of a negative control (secondary antibody alone) in order to evaluate the specificity of the antibodies. Three different states of microglia activation, ranging from resting to intermediate and fully activated according to their morphological appearance, were determined and then quantified by a rater blinded to the treatment group. Resting microglial cells have a ramified morphology with a relatively small body. Intermediate activated microglial cells display a strong expression of CD11b, shortening processes. Fully activated microglial cells adopt a round, macrophage-like morphology. Quantification of microglia was made by manually counting CD11b^+^ cells in the ventral horn of gray matter to include layers 7 to 9 of the spinal cord at 400× magnifications.

### Spinal cord TNFα ELISA

Wild-type control mice, hMSC-treated and vehicle-treated transgenic mice at 11 weeks of age (four mice each group) were killed by decapitation, and the lumbar spinal cord was rapidly removed and frozen in isopentane at −35°C on dry ice. Spinal cord samples were placed in ice-cold lysis buffer containing protease inhibitor, homogenized using a homogenizer, and centrifuged at 13,000× g for 30 minutes at 4°C. Protein concentrations from supernatant were determined using a Bio-Rad protein assay (Bio-Rad, Hercules, CA, USA ). The supernatant was removed and stored in −80°C in aliquots for ELISA and western blotting studies. TNFα concentrations were determined by a mice-specific ELISA kit (BioSource International, Camarillo, CA, UAS) according to the manufacturer’s instructions.

### Western blotting

Briefly, protein extracts (20 μg) of the lumbar enlargement were boiled in loading buffer for 3 minutes and were subsequently loaded into wells of 12% SDS-polyacrylamide gels at 80 V for 30 minutes and at 150 V for 90 minutes. Proteins were then transferred onto polyvinylidene difluoride membranes (Minipore, Bedford, MA, USA). The blots were blocked with 5% (w/v) defatted milk in TBS-Tween20 (pH 8.0) for 1 hour at room temperature and then incubated overnight at 4°C with primary antibodies: polyclonal rabbit anti-mouse inducible nitric oxide synthase (iNOS) (1:1,000; Santa Cruz Biotechnology, Santa Cruz, CA, USA) and mouse anti-actin (1:1,000; Santa Cruz Biotechnology). The blots were washed three times in TBS-Tween20 for 10 minutes each, incubated with a horseradish peroxidase-conjugated goat anti-rabbit IgG (1:5,000; Chemicon) or a horseradish peroxidase-conjugated goat anti-mouse IgG (1:2,000; Chemicon) for 1 hour at room temperature, and washed four times in TBS-Tween20 for 10 minutes each. Protein bands were visualized after incubation with SuperSignal West Pico chemiluminescent substrate (Pierce Biotechnology, Rockford, IL, USA) and exposure of high-performance chemiluminescence film to the membrane surface in the dark. Quantitative analysis of the intensity of the bands was performed with Band Scan 5.0 software (Glyco, San Leandro, CA, USA).

### Primary microglia cultures

Primary microglial cells were cultured from newborn C57/BL6 mice by a method described previously [16]. In brief, the meninges were removed from the forebrains, and tissues collected from forebrains were triturated into single cells using fire-polished long Pasteur pipettes in DMEM with high glucose. Cells were plated onto a poly-l-lysine (Sigma)-coated (10 μg/ml) flask (75 cm^2^) in media with 10% FBS and incubated at 37°C in 5% CO_2_ in air and 95% humidity. The culture medium was replenished after 24 hours and incubated for 8 days. Microglial cells were then purified from the initial mixed culture by sequential shaking at 180 ×g for 30 minutes at room temperature. The resultant supernatant was collected and centrifuged at 150× *g* for 5 minutes. The pellet was suspended in complete medium and then plated in 24-well culture plates (Falcon, Franklin Lakes, NJ, USA). After 1 hour, microglial cells were further purified by washing twice with serum-free media and grown in new complete media. To determine the purity of the microglial cells, immunocytochemical analysis was carried out using rat anti-mouse F4/80 antibody (1:50; Serotec). These cultures were >95% F4/80 positive as determined by immunocytochemistry, indicating that they were composed of microglial cells.

### Treatment

Microglia were treated with either hMSC co-culture in which two cells interacted through contact, hMSC conditioned media in which two-cell interaction is not available, or hMSC transwell culture in which two cells interacted through diffusible molecules only.

Microglial cells were randomly divided into four groups. Group 1, co-culture group: for co-culture plating, microglia and hMSCs were mixed at a density of 1×10^5^ cells/ml for microglia and 5×10^4^ cells/ml for hMSCs in DMEM with high glucose and 10% FBS. Then 1 ml of the cell suspension was plated in 24-well plates (Falcon) and allowed to settle for 24 hours at 37°C in 5% CO2 in air.

Group 2, transwell culture group: 1×10^5^ microglia per well were plated in 1 ml DMEM with high glucose and 10% FBS in 24-well plates, and allowed to settle for 3 hours at 37°C in 5% CO_2_ in air and 95% humidity. In a separate plate, 5×10^4^ hMSCs per insert were plated in 300 μl DMEM with high glucose and 10% FBS in cell culture inserts (1 μm pore size; Falcon), and allowed to settle at 37°C in 5% CO_2_ in air. Cell culture inserts containing the hMSCs were then inserted into the wells containing the microglia and incubated for a further 21 hours before the start of the experiment.

Group 3, conditioned medium group: 2×10^5^ hMSCs were plated in 25 cm^2^ flask in 5 ml DMEM with high glucose and 10% FBS. The culture medium was replenished after 24 hours and incubated for 48 hours. The resultant supernatant was collected and centrifuged at 150× *g* for 5 minutes to remove cellular debris as hMSC conditioned medium. Then 1×10^5^ microglia per well were plated in 1 ml medium in 24-well plates and allowed to settle for 24 hours at 37°C in 5% CO_2_ in air before addition of hMSC conditioned medium. Microglial medium was removed and replaced with 1 ml hMSC conditioned medium. Microglia were incubated for a further 24 hours at 37°C in 5% CO_2_ in air.

Group 4, control group: 1×10^5^ microglia were plated in 24-well plates in 1 ml DMEM with high glucose and 10% FBS. Microglial cells were stimulated with 1 μg/ml lipopolysaccharide (LPS; Sigma Chemical, St Louis, MO, USA) for 12 hours based on the literature. The experiments were carried out in triplicate and each experiment was repeated three times.

### Immunocytochemistry

The microglial cells were fixed with 4% paraformaldehyde in PBS (pH 7.4) for 20 minutes, blocked with normal serum for 1 hour, and then incubated with primary antibody at 4°C overnight. Primary antibodies included: rat anti-mouse F4/80 (1:50; Serotec), and mice anti-human HuNu (1:50; Chemicon). Cells were washed and incubated at room temperature for 1 hour with goat anti-rat FITC-conjugated IgG (1:50; Serotec) and goat anti-mice Cy3-conjugated IgG (1:200; Chemicon). These cells were counterstained with 4′,6-diamidino-2-phenylindole.

### Statistical analysis

Kaplan–Meier survival analysis and log-rank (Mantel–Cox) analysis were used for survival comparisons. All other statistical analyses were performed by one-way analysis of variance followed by the Tukey *post hoc* test. All group values are expressed as mean ± standard error of the mean.

## Results

### Human marrow stromal cell character

The cells isolated from bone marrow were hMSCs showing the specific features defined by the International Society for Cellular Therapy guidelines [17]. In fact, they were adherent cells positive (≥95%) for CD73, CD90 and CD105 and negative (≤2%) for the other markers. Moreover, these cells were able to differentiate to osteoblasts, chondroblasts and adipocytes after exposure to specific conditioning media, as previously shown [18].

### Human marrow stromal cell transplantation delayed disease progression and prolonged survival in SOD1 mice

**Figure 1:**
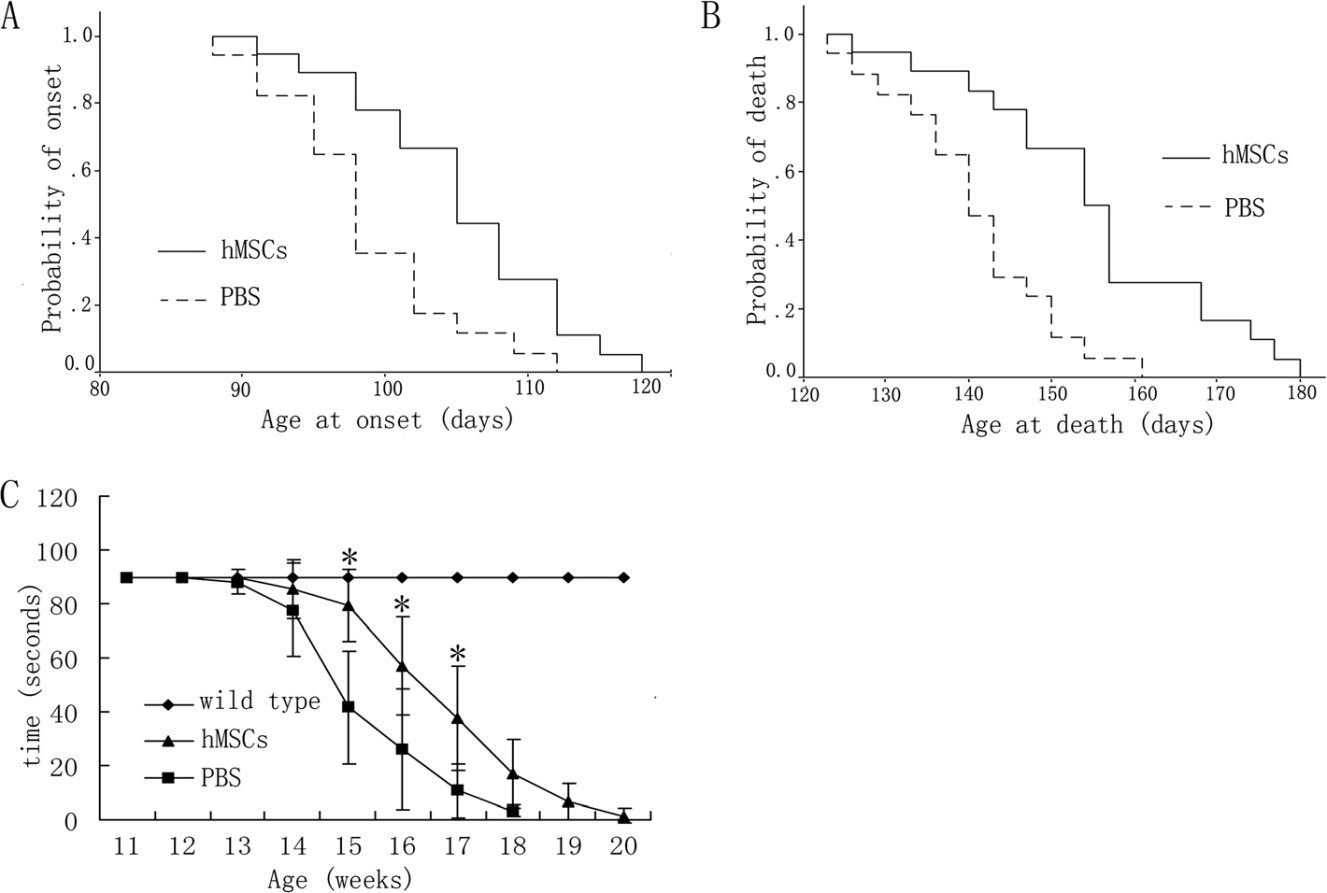
**Human marrow stromal cell transplantation delayed disease progression and prolonged survival in SOD1 mice.** Cumulative probability of disease onset (**A**) and survival (**B**) for Cu/Zn superoxide dismutase 1 (SOD1) mice beginning at 8 weeks of age treated with intrathecal injection of human marrow stromal cells (hMSCs), relative to PBS treatment. Survival and onset of SOD1 mice were significantly improved by hMSC treatment (*P <0.01). (**C**) Effect of hMSC treatment on motor performance in G93A SOD1 transgenic mice from 8 weeks of age. There was a significantly improved motor performance in G93A mice treated with hMSC injection from 15 to 17 weeks of age (*P <0.01). Values are mean ± standard error of the mean.

We investigated the therapeutic effect of intrathecally transplanted hMSCs in G93A mice. As compared with vehicle-treated SOD1 mice, hMSC-treated animals showed a 6-day increase in median onset (104.8 ± 8.5 days vs. 98.7 ± 5.9 days, *χ*^*2*^ = 8.61, *P* <0.01; Figure 1A), and mean survival of hMSC-treated animals was prolonged by 14 days (154.82 ± 15.16 days vs. 140.47 ± 10.94 days, *χ*^*2*^ = 10.13, *P* <0.01; Figure 1B).

Assessment of neuromuscular function was performed by quantitative grip strength. SOD1 transgenic mice typically manifest a decline in motor performance after clinical onset. Consistent with the finding of clinical observation, hMSC-transplanted mice had significantly better motor performance from 15 to 17 weeks of age than vehicle-treated mice (*P* <0.01; Figure 1C).

### Attenuation of neuronal loss by intrathecal administration of human marrow stromal cells

**Figure 2:**
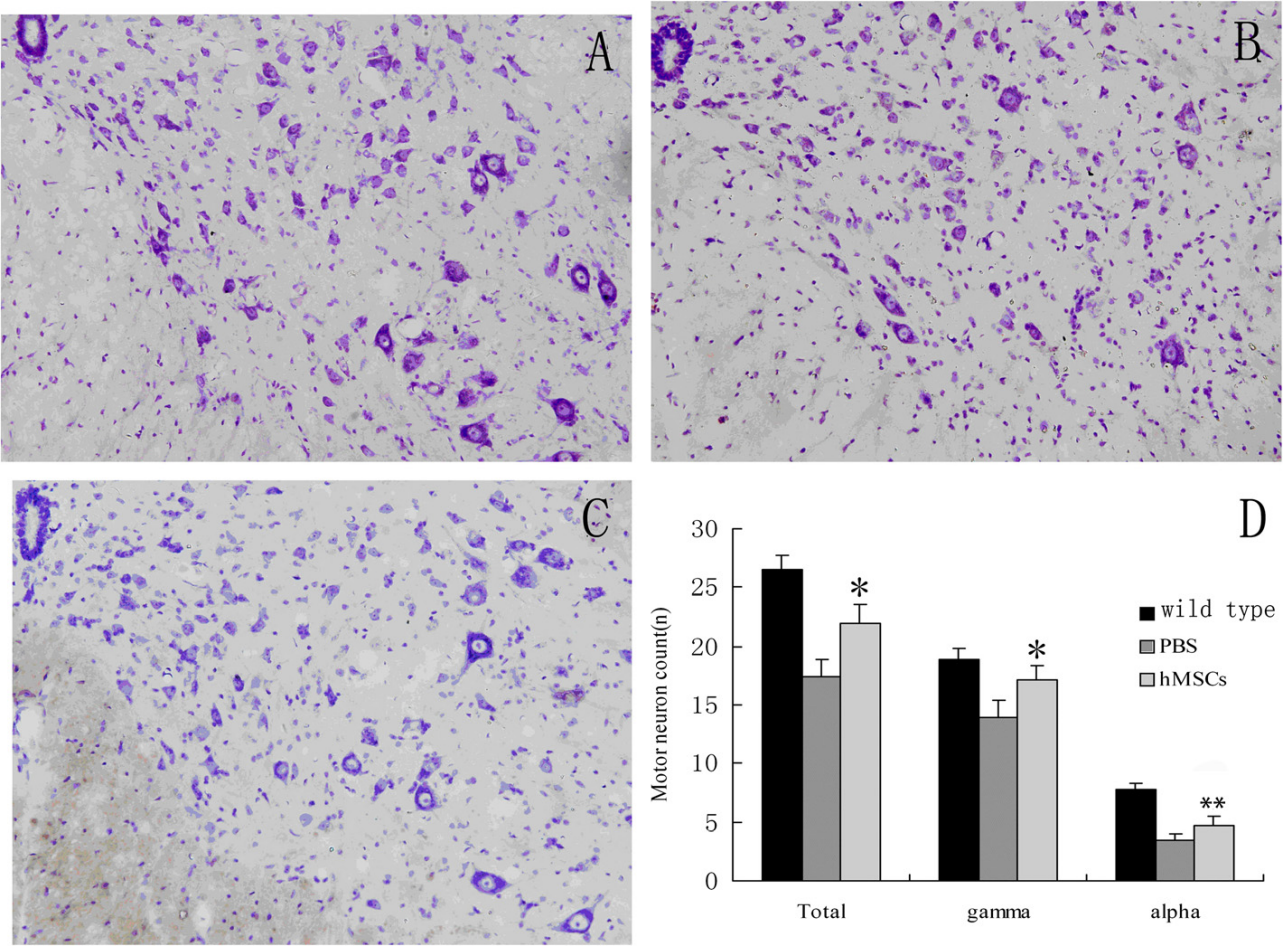
**Attenuation of neuronal loss by intrathecal administration of human marrow stromal cells.** Cresyl violet staining of the lumbar spinal cord sections from 15-week-old wild-type mice (**A**) and Cu/Zn superoxide dismutase 1 (SOD1) transgenic mice treated with PBS (**B**) and human marrow stromal cells (hMSCs) (**C**) at 200× magnification. (**D**) Quantitative cell counts of motor neurons in the anterior horn sections. Intrathecally injected hMSCs significantly preserved motor neuron loss in SOD1 transgenic mice at the age of 15 weeks (*P <0.01and **P <0.05 versus PBS alone).

We examined motor neurons in the lumbar spinal cord of wild-type mice, hMSC-treated and vehicle-treated transgenic mice at the age of 11 and 15 weeks by Nissle staining (Figure 2A, B, C). Average motoneuron counts per section in hMSC-treated mice were similar to counts in vehicle-treated mice (23.6 ± 1.2 vs. 22.7 ± 1.7; *P* >0.05) at the age of 11 weeks, whereas hMSC transplantation significantly preserved motoneurons compared with vehicle-treated mice at the age of 15 weeks (21.9 ± 1.6 vs. 17.4 ± 1.5; *P* <0.01; Figure 2D). The most vulnerable motoneurons in ALS, which are the alpha neurons, were also significantly preserved in hMSC-treated mice at the age of 15 weeks (4.7 ± 0.7 vs. 3.5 ± 0.4; *P* <0.05; Figure 2D).

### Inhibition of microglial activation and inflammatory factors production by human marrow stromal cell treatment

**Figure 3:**
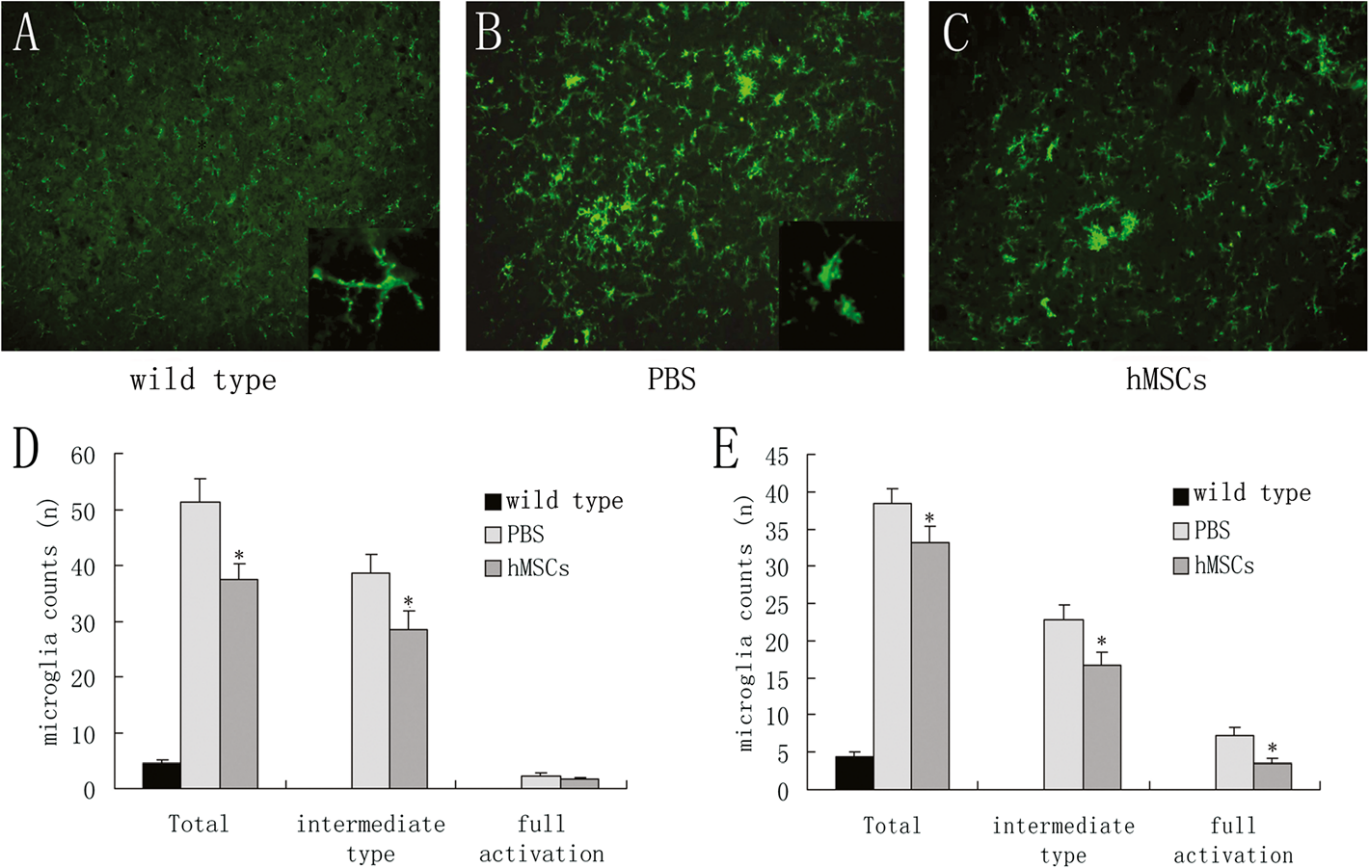
**Inhibition of microglial activation by human marrow stromal cell treatment in SOD1 transgenic mice.** Activation of microglia were detected by CD11b immunoreactivity in the spinal cord ventral gray of wild-type mice (**A**), PBS-treated transgenic mice (**B**), and human marrow stromal cell (hMSC)-treated transgenic mice (**C**) at the age of 15 weeks at 400× magnification. Higher-magnification images are shown in the insert for resting microglia (A) and activated microglia (B). Quantitative cell counts of CD11b-positive microglia in the ventral gray of mice at 11 weeks of age (**D**) and 15 weeks of age (**E**). Intrathecally injected hMSCs significantly suppressed microglial activation in Cu/Zn superoxide dismutase 1 (SOD1) transgenic mice (*P <0.01 versus PBS alone).

Activation of microglia was examined by CD11b immunohistochemistry in the spinal cord ventral grey of wild-type mice and transgenic mice at the age of 11 and 15 weeks (Figure 3A, B, C). Few microglia cells were observed in wild-type mice at the age of 11 weeks (4.5 ± 0.6). However, we found a substantial increase in the number of CD11b-positive microglia in transgenic mice at 11 weeks (51.3 ± 4.2) and 15 weeks (38.4 ± 3.6) of age in SOD1 transgenic mice. Three different states of microglial activation, ranging from resting to intermediate and fully activated, were determined and then quantified. Notably, hMSC injection significantly reduced the number of total microglial cells and activated microglia in SOD1 transgenic mice at the ages of 11 weeks and 15 weeks (*P* <0.01; Figure 3D, E).

**Figure 4:**
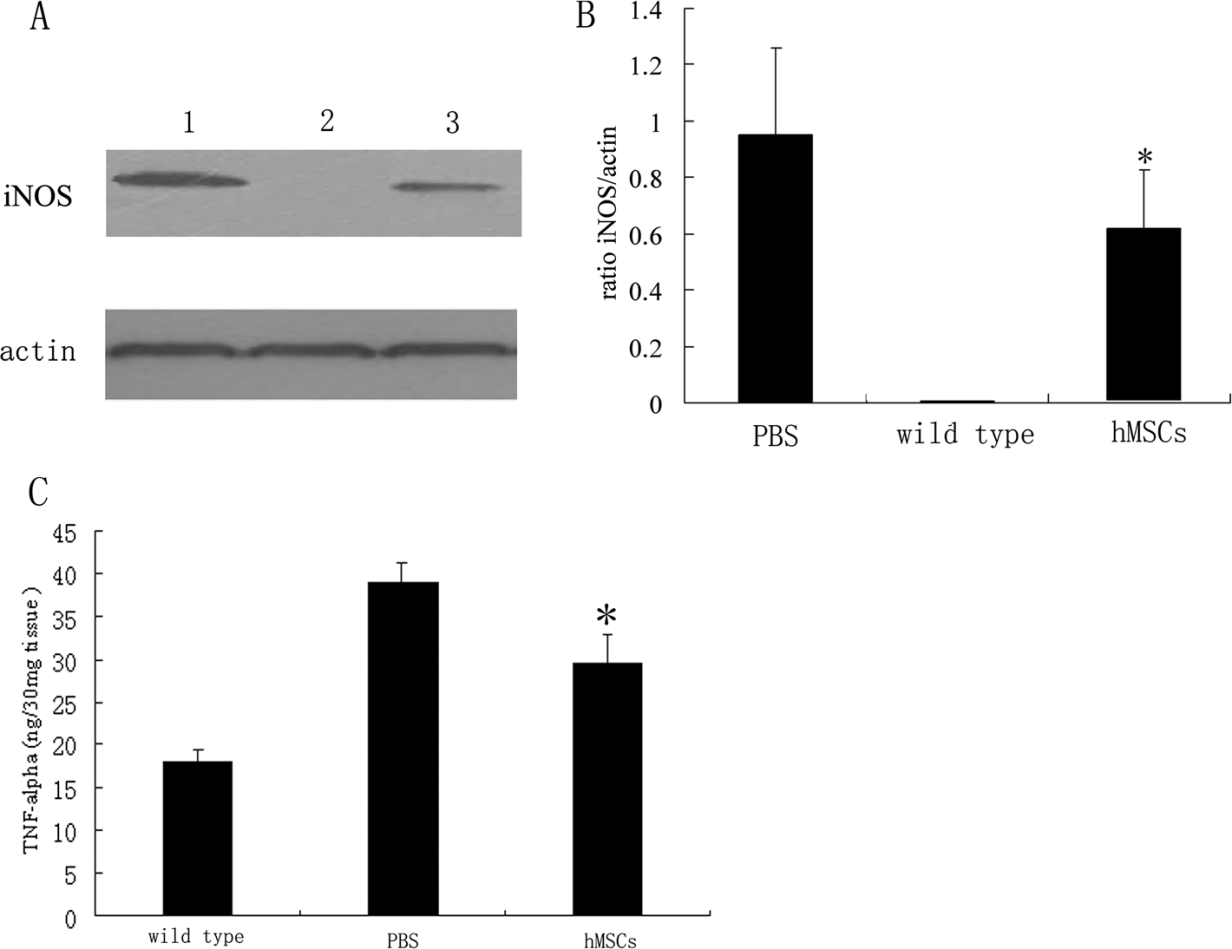
**Inhibiting TNFα and iNOS production by human marrow stromal cell treatment in SOD1 transgenic mice.** Lumbar spinal cord tissues from wild-type mice, PBS-treated and human marrow stromal cell (hMSC)-treated transgenic mice at the age of 11 weeks are assayed for inducible nitric oxide synthase (iNOS) protein expression by Immunoblot and TNFα level by ELISA. (**A**) Representative immunoblots for iNOS and β-actin of the lumbar spinal cord from PBS-treated transgenic mice (1), wild-type mice (2) and hMSC-treated transgenic mice (3). (**B**) Quantitative evaluation through optical densitometry of iNOS blot and relation to β-actin blot. The levels of iNOS in Cu/Zn superoxide dismutase 1 (SOD1) transgenic mice receiving hMSC transplantation were significantly reduced compared with those in vehicle-treated mice (*P <0.01 versus PBS alone). (**C**) Intrathecally injected hMSCs significantly decreased the spinal cord TNFα level in SOD1 transgenic mice (*P <0.01 versus PBS alone).

Activated microglia secrete various inflammatory molecules including TNFα and nitric oxide, so we examined the protein levels of TNFα and iNOS of lumbar spinal cord from wild-type mice and transgenic mice. Immunoblot analysis of iNOS in spinal cord extracts from 11-week-old hMSC-treated and vehicle-treated transgenic mice revealed a single band of about 130 kDa, and in contrast no signal in wild-type mice (Figure 4A). The levels of iNOS in SOD1 transgenic mice receiving hMSC transplantation were lower by about 31% than those in vehicle-treated mice (Figure 4B; *P* <0.01). Levels of TNFα were significantly elevated in vehicle-treated SOD1 mice at the age of 11 weeks compared with normal control mice (Figure 4C; *P* <0.01). In accord with reduced microglial activation, administration of hMSCs significantly reduced the levels of TNFα by about 24% in transgenic mice at the age of 11 weeks (Figure 4C; *P* <0.01).

### Inhibition of microglial activation by human marrow stromal cells *in vitro*

**Figure 5:**
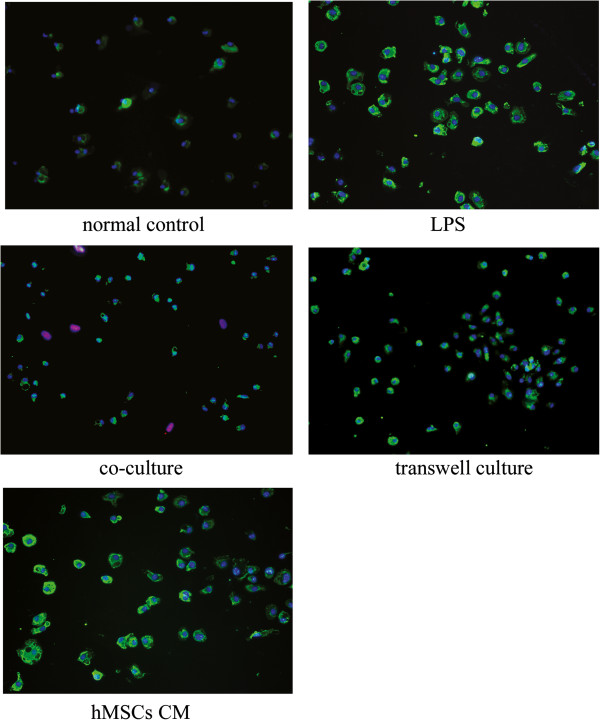
**Effects of human marrow stromal cells on lipopolysaccharide-induced activation of microglia.** Microglia were stimulated with 1 mg/μl lipopolysaccharide (LPS), and cultures were treated with human marrow stromal cells (hMSCs) in co-cultures and transwell cultures, and hMSC conditioned medium (CM) (400× magnifications). Cells were fixed after 12 hours of stimulation with LPS. Microglial cells were immunostained with an antibody against the F4/80 antigen, and hMSCs were stained with anti-HuNu antibody. In normal control cultures, the majority of microglial cells were small and round. Upon stimulation with LPS, most of the microglial cells showed an enlarged shape. MSCs in co-cultures and transwell cultures dramatically inhibited microglial activation, whereas hMSC CM has no significant effect on the microglial activation.

To investigate the mode of hMSCs inhibiting microglial activation, microglia were treated with either hMSC co-culture in which two cells interacted through contact, hMSC transwell culture in which two cells interacted through diffusible molecules only, or hMSC conditioned medium in which two-cell interaction is not available. Activated microglia stimulated with LPS showed an enlarged shape and an intense immunoreactivity for F4/80 antigen (Figure 5B), whereas untreated microglia in cultures showed a round shape (Figure 5A). Most of the microglia in hMSCs-microglia co-cultures (Figure 5C) and transwell cultures (Figure 5D) stimulated by LPS were small and round, similar to resting state. This indicated that the microglial cells were not fully activated by LPS in the presence of hMSCs. However, hMSC conditioned medium failed to suppress the microglia activation (Figure 5E).

## Discussion

In this study, we suggest for the first time that intrathecally transplanted hMSCs can inhibit the inflammatory response to exert a neuroprotective effect in SOD1 transgenic mice. This is evidenced by the decreases in microglial activation, TNFα secretion and iNOS protein expression. In addition, our study demonstrates that the inhibitory effect on microglial activation of hMSCs is through secretion of diffusible molecules adjusted to environmental cues.

Microglia play a critical role as resident immunocompetent and phagocytic cells within the central nervous system. Activation is associated with increased nitric oxide, reactive oxygen species and proinflammatory cytokines, such as TNFα and IL-1β, which could generate a neuroinflammatory environment. There is little doubt that activated microglia can inflict significant damage on neurons [19]. Targeting therapy to microglia may therefore be an effective strategy for ALS. Recently, the anti-inflammatory effect of MSCs has generated a great deal of interest. *In vitro* studies have shown that MSCs can regulate immune cell proliferation, differentiation and phenotype [8-10]. The anti-inflammatory properties of MSCs have been well demonstrated in the treatment of graft-versus-host disease after allogeneic hematopoietic cell transplantation, and phase Ш clinical trials are currently ongoing [9]. In animal models of Parkinson disease [11], cerebral ischemia [12,13], Krabbe’s disease [14] and ALS [15], studies suggested that administration of MSCs attenuated inflammation and improved the motor function compared with control animals. hMSC transplantation in transient global ischemic mice downregulated >10% of the ischemic-induced genes, most of which were involved in inflammatory and immune responses [12]. These studies suggest that the modulation of inflammatory and immune responses may be one underlying mechanism of the neuroprotective effect of MSCs. Our study showed that hMSCs injected intrathecally had an inhibitory effect on the microglial activation and attendant inflammatory molecule production, which is consistent with previous reports. Interestingly, we observed the attenuation of neuronal loss by hMSC treatment in the later period of SOD1 mice (15 weeks). In contrast, the inhibitory effect on microglial activation and inflammatory molecules production occurred in the early period of SOD1 mice (11 weeks). Strong evidence accumulated over recent years indicates that microglial cells could be involved in the initiation and propagation of motor neuronal cell damage in ALS [19]. Microglial activation played an important role in later disease progression, but not in disease onset and an early phase of disease progression in SOD1 mice [6]. The neuroprotective effect of hMSCs may thus be a consequence of the inhibition of microglial activation that occurs in the early stages. Our results therefore suggest that the neuroprotective effect of hMSCs is consistent with the anti-inflammation mechanism. It is well known that neurons can inhibit microglial activation, but damaged neurons can activate resting microglial cells [20]. hMSCs seem to have a similar effect on microglial activation as normal neurons, which may provide a novel concept for cytotherapy.

To further investigate the mode of hMSCs inhibiting microglial activation, primary microglial cells were treated with hMSC co-culture, hMSC transwell culture, or hMSC conditioned medium in our studies. Our results suggested that hMSCs reduced microglial activation through either co-culture or transwell culture, which indicates that hMSCs can inhibit microglial activation through diffusible soluble factors. The mode of hMSCs inhibiting microglial activation through transwell culture provides important evidence for our findings that intrathecal administration of hMSCs may reduce microglial activation in SOD1 mice. Intrathecal injection is a potential and practical route of cell transplantation for central nervous system disease because of its minimal invasiveness and the fact that it would bypass the blood–brain barrier. Whether intrathecally injected cells can migrate into the parenchyma of the spinal cord is still under debate [21-25]. The difference in cell integration may be attributable to the animal models and transplanted cells. The pial matter might act as a barrier to invasion of cells into the spinal cord parenchyma, so the injured pial layer in some animal models may allow cells to more easily invade the spinal cord parenchyma. Additionally, various cells have different migration behavior. Habisch and colleagues showed that few intrathecally injected hMSCs can integrate the spinal cord parenchyma in SOD1 mice [25], which is consistent with our previous study [5]. Encouragingly, studies found that intrathecally transplanted cells were transported extensively by cerebrospinal fluid within the subarachnoid space, survived well and even proliferated on the pial surface of the spinal cord [21,23,24]. Thus it would not be unexpected that the transplanted cells remaining in cerebrospinal fluid are responsible for the neuroprotective effects through secreting some diffusible soluble factors to inhibit microglial activation.

Mechanisms by which MSCs inhibit microglial activation are still under investigation. The secretion of various cytokines and neurotrophic factors is now believed to be the main mechanism by which MSCs achieve their therapeutic effects [10]. Studies have shown that hMSCs have the anti-inflammation effects involving the secretion of prostaglandin E_2_, human leukocyte antigen G5, hepatocyte growth factor, iNOS, indoleamine-2,3-dioxygenase, transforming growth factor beta, leukemia-inhibitory factor, IL-10, vascular endothelial growth factor and insulin-like growth factor [10]. In addition, our study showed that hMSC co-culture can also inhibit microglial activation. Non-neural cells extend the survival of SOD1 mutant motor neurons in SOD1 chimeric mice, which correlates well with the proportion of wild-type cells within the spinal cord [26]. The study suggests that it may be feasible to achieve a more efficient therapeutic effect by enhancing entry of transplanted cells into the spinal cord parenchyma. The study of the method by which the invasion of cells into the lesions can be promoted is therefore necessary. Interestingly, conditioned medium from hMSCs has no effect on microglial activation, which suggests that hMSCs exert an anti-inflammation effect through a positive process. Studies showed that MSCs respond to certain cytokines such as IFNγ, TNFα, IL-1α and IL-1β that could be present in the local environment [10]. MSCs may therefore possibly produce diffusible soluble factors adjusted to the needs of tissue, which may be their most important biological property.

## Conclusions

How cells injected intrathecally exert a neuroprotective effect is still unclear, because very few injected hMSCs were detected in the parenchyma of the spinal cord. Understanding the mechanism of cell therapy will assist in the improvement of therapeutic efficacy. Our current study shows that intrathecally injected hMSCs can attenuate the microglial activation through secretion of diffusible molecules to exert a therapeutic effect in SOD1 transgenic mice, which provides important evidence for cell transplantation through cerebrospinal fluid. Further studies are needed to explore the exact mechanism by which hMSCs inhibit inflammation to facilitate the therapeutic effect.

## Abbreviations

ALS: Amyotrophic lateral sclerosis; DMEM: Dulbecco’s modified Eagle’s medium; ELISA: Enzyme-linked immunosorbent assay; FBS: Fetal bovine serum; FITC: Fluorescein isothiocyanate; hMSC: Human marrow stromal cell; IFN: Interferon; IL: Interleukin; iNOS: Inducible nitric oxide synthase; LPS: Lipopolysaccharide; MSC: Marrow stromal cell; PBS: Phosphate-buffered saline; PCR: Polymerase chain reaction; SOD1: Cu/Zn superoxide dismutase 1; TNF: Tumor necrosis factor

## Competing interests

The authors declare that they have no competing interests.

## Authors’ contribution

Chang Zhou carried out cell culture, immunohistochemistry and drafted the manuscript. RLZ carried out ELISA and western blotting. Chen Zhang provided substantial contributions to the concept and design of the study and manuscript draft. SC contributed to animal observation and the statistical analysis. PG contributed to image acquisition, technical and conceptual study elements. Cheng Zhang contributed to the concept and design of the study, interpretation of data and manuscript revision. All authors read and approved the final manuscript.
